# Supplementary material for: Interaction of preimplantation factor with the global bovine endometrial transcriptome
Source: PLoS One. 2020 Dec 7;15(12):e0242874. doi: 10.1371/journal.pone.0242874 (PMC7721156; doi:10.1371/journal.pone.0242874)

S1 Fig. PCA plot showing all RNA sequencing replicates prior to the two technical replicates for cows 5-7 being summed together. Variance was evident between the samples on each lane (1 and 2), but not between the technical replicates (2a and 2b) of cows 5-7 which were sequenced twice to ensure similarity in the number of reads between all samples. The first two principle components are displayed.

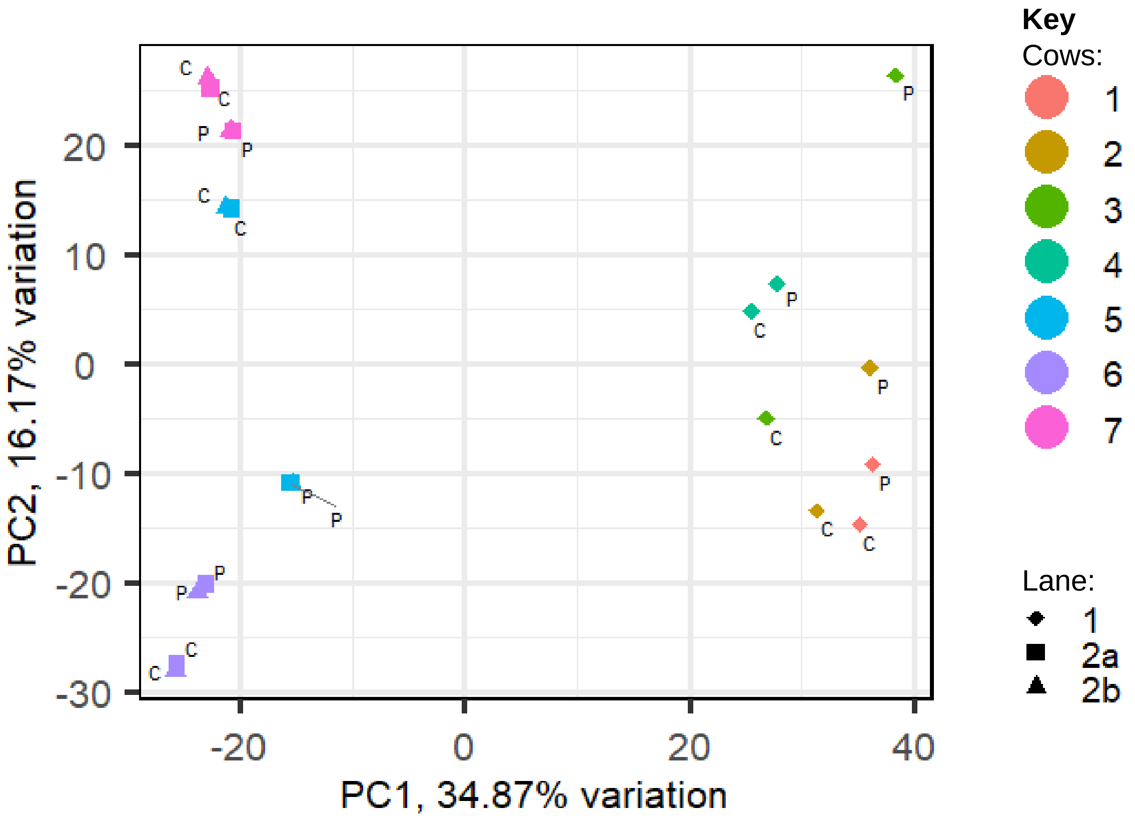

Supplement: S1 Fig — Variance was evident between the samples on each lane (1 and 2), but not between the technical replicates (2a and 2b) of cows 5–7 which were sequenced twice to ensure similarity in the number of reads between all samples. The first two principle components are displayed. (PDF) [file pone.0242874.s001.pdf]
