# Supplementary material for: Interaction of preimplantation factor with the global bovine endometrial transcriptome
Source: PLoS One. 2020 Dec 7;15(12):e0242874. doi: 10.1371/journal.pone.0242874 (PMC7721156; doi:10.1371/journal.pone.0242874)

S2 Fig. PCA plots demonstrating principle components 1 – 4. Variances were detected between animal replicates and samples treated with or without sPIF (100nM). The plot demonstrating principle component 1 and 2 is located in Fig 1b.

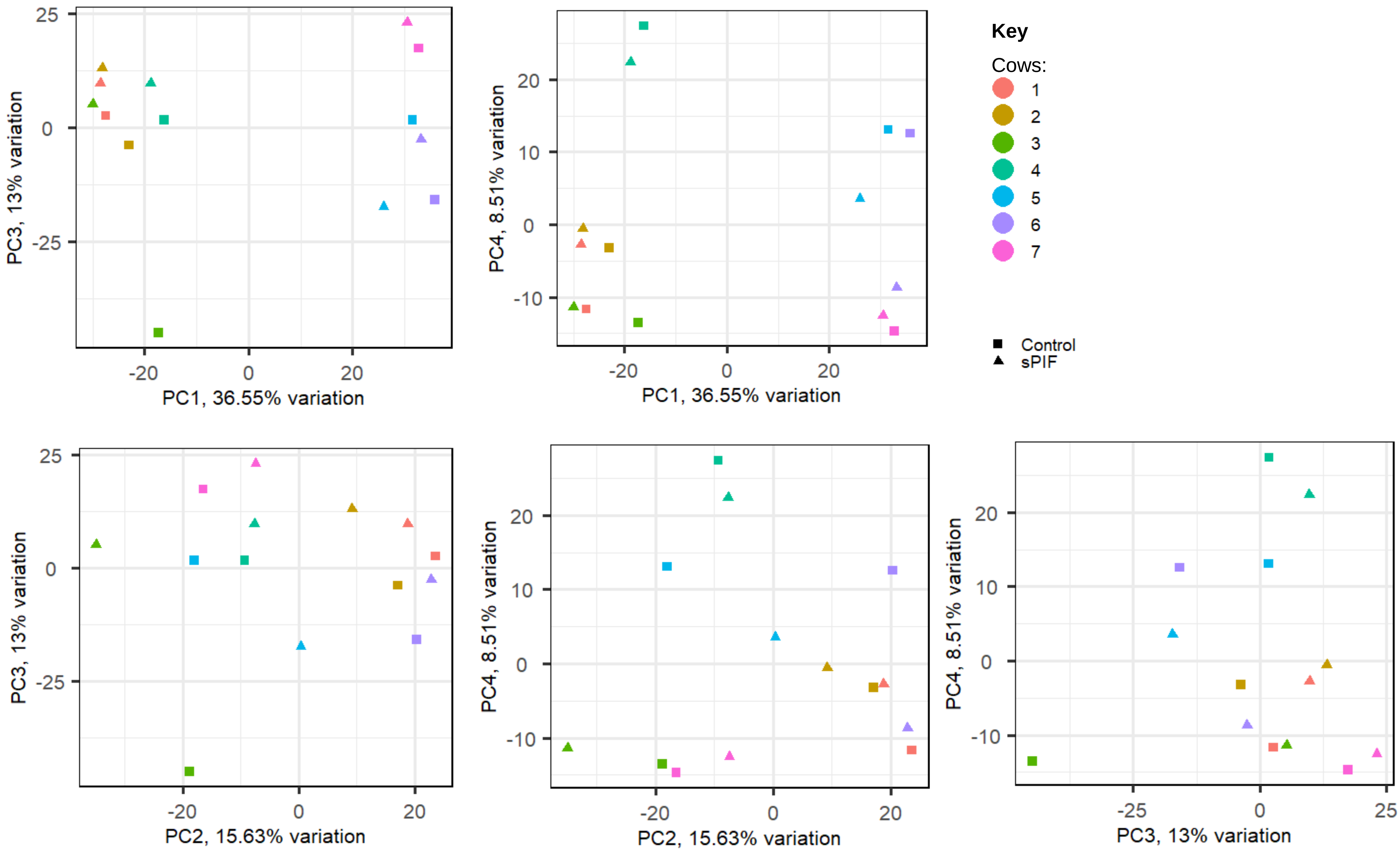

Supplement: S2 Fig — Variances were detected between animal replicates and samples treated with or without sPIF (100nM). The plot demonstrating principle component 1 and 2 is located in Fig 1B. (PDF) [file pone.0242874.s002.pdf]
