# Supplementary material for: Interaction of preimplantation factor with the global bovine endometrial transcriptome
Source: PLoS One. 2020 Dec 7;15(12):e0242874. doi: 10.1371/journal.pone.0242874 (PMC7721156; doi:10.1371/journal.pone.0242874)
Supplement: S1 Table — Based on P adjusted values (Padj<0.1) as assessed by the Bioconductor package, deSeq2 statistical analysis. (PDF) [file pone.0242874.s003.pdf]

S1 Table. Differentially expressed genes (DEG) following sPIF treatment of the bovine endometrium, compared to the control. Based on P adjusted values (*P*adj<0.1) as assessed by the Bioconductor package, deSeq2 statistical analysis.

Gene iD	log2FoldChange	padj	Gene name	Description - ensembl
ENSBTAG00000013705	-0.63517	0.0001	NFKBIE	NFKB inhibitor epsilon
ENSBTAG00000012178	-0.86986	0.000336	NR1D1	nuclear receptor subfamily 1 group D member 1
ENSBTAG00000012343	-0.76812	0.003689	TSPAN5	tetraspanin 5
ENSBTAG00000020270	-0.57265	0.004743	NFKB1	nuclear factor kappa B subunit 1
ENSBTAG00000031231	-0.76721	0.005789	IRF1	interferon regulatory factor 1
ENSBTAG00000011207	-0.68419	0.008343	CNN1	calponin 1
ENSBTAG00000005280	-0.67475	0.010615	ADA	adenosine deaminase
ENSBTAG00000004305	-0.48984	0.010615	RGS16	regulator of G protein signaling 16
ENSBTAG00000007773	-1.20392	0.014501	VCAM1	vascular cell adhesion molecule 1
ENSBTAG00000004036	-0.71393	0.014501	GJC1	gap junction protein gamma 1
ENSBTAG00000032534	-0.40484	0.015062	RHOF	ras homolog family member F, filopodia associated
ENSBTAG00000046971	-0.47058	0.017626	novel gene	
ENSBTAG00000020620	-0.63608	0.018348	novel gene	
ENSBTAG00000000283	-0.76363	0.02042	CSF1	colony stimulating factor 1
ENSBTAG00000007239	-0.79912	0.022772	TNFAIP6	TNF alpha induced protein 6
ENSBTAG00000010497	-0.38376	0.032211	TRAF2	TNF receptor associated factor 2

S1 Table

ENSBTAG00000021526	-0.78926	0.033947	RPRM	reprimo, TP53 dependent G2 arrest mediator homolog
ENSBTAG00000048062	-0.45288	0.03439	KDM6B	lysine demethylase 6B
ENSBTAG00000003880	-0.80597	0.037178	EMILIN2	elastin microfibril interfacer 2
ENSBTAG00000018013	-0.62773	0.037178	EMP3	epithelial membrane protein 3
ENSBTAG00000003033	-0.54209	0.037178	GADD45G	growth arrest and DNA damage inducible gamma
ENSBTAG00000010303	-0.68479	0.038776	novel gene	
ENSBTAG00000039657	-0.70937	0.043413	HIST1H2AC	histone cluster 1 H2A family member c
ENSBTAG00000009496	-0.43262	0.049669	STAT5A	signal transducer and activator of transcription 5A
ENSBTAG00000031430	-0.86848	0.050257	CD83	CD83 molecule
ENSBTAG00000001027	-0.7483	0.050257	SERPINH1	serpin family H member 1
ENSBTAG00000034633	-0.60669	0.050257	ERO1B	ERO1-like protein beta precursor
ENSBTAG00000000436	-0.43907	0.050257	TNFAIP3	TNF alpha induced protein 3
ENSBTAG00000011424	-0.42482	0.050257	TPM2	tropomyosin 2 (beta)
ENSBTAG00000017508	-0.85547	0.051184	CYSLTR2	cysteinyl leukotriene receptor 2
ENSBTAG00000005998	-0.40018	0.051476	NOCT	nocturnin
ENSBTAG00000016874	-0.95082	0.054949	DNAJB1	DnaJ heat shock protein family (Hsp40) member B1
ENSBTAG00000014790	-0.52389	0.054949	ZBTB2	zinc finger and BTB domain containing 2

S1 Table

ENSBTAG00000020736	-0.40052	0.054949	CD40	CD40 molecule
ENSBTAG00000015212	-0.39836	0.055857	IFNAR2	interferon alpha and beta receptor subunit 2
ENSBTAG00000037778	-0.70621	0.05625	novel gene	
ENSBTAG00000020755	-1.82879	0.057043	SELP	selectin P
ENSBTAG00000012212	-1.1176	0.057043	CYP26B1	cytochrome P450 family 26 subfamily B member 1
ENSBTAG00000043250	-0.54999	0.057043	7SK	7SK RNA
ENSBTAG00000023601	-0.45077	0.057043	CD3EAP	CD3e molecule associated protein
ENSBTAG00000013577	-0.31241	0.057043	SLC1A5	solute carrier family 1 member 5
ENSBTAG00000045858	-1.28803	0.062945	DNAJB1	DnaJ heat shock protein family (Hsp40) member B1
ENSBTAG00000042377	-1.26227	0.062945	SNORD33	Small nucleolar RNA Z195/SNORD33/SNORD32 family
ENSBTAG00000014912	-1.04395	0.062945	FMOD	fibromodulin
ENSBTAG00000003938	-0.86198	0.062945	FNDC1	fibronectin type III domain containing 1
ENSBTAG00000008483	-0.5502	0.062945	CALCRL	calcitonin receptor like receptor
ENSBTAG00000048228	-0.45696	0.062945	novel gene	
ENSBTAG00000017560	-0.34219	0.062945	CEBPG	CCAAT/enhancer binding protein gamma
ENSBTAG00000037539	-1.19705	0.065335	novel gene	
ENSBTAG00000003253	-0.82074	0.074624	NPPC	natriuretic peptide C
ENSBTAG00000047676	-1.00733	0.075279	novel gene	
ENSBTAG00000046218	-0.35543	0.075279	KLF11	Kruppel like factor 11
ENSBTAG00000012855	-0.95058	0.076909	LPL	Bos taurus lipoprotein lipase (LPL), mRNA
ENSBTAG00000002615	-0.56713	0.077332	LONrf3	LON peptidase N-terminal domain and ring finger 3

S1 Table

ENSBTAG00000010322	-0.61103	0.078199	HYOU1	hypoxia up-regulated protein 1 precursor
ENSBTAG00000013131	-0.63791	0.078566	FAM110D	family with sequence similarity 110 member D
ENSBTAG00000013066	-0.63069	0.080742	IGF2	Insulin-like growth factor II Insulin-like growth factor II Prepti
ENSBTAG00000011146	-0.42251	0.080742	RAB8B	RAB8B, member RAS oncogene family
ENSBTAG00000011912	-0.26572	0.080742	SLC25A25	solute carrier family 25 member 25
ENSBTAG00000002781	-0.78357	0.084836	GCNT4	glucosaminyl (N-acetyl) transferase 4, core 2
ENSBTAG00000003100	-0.43445	0.086277	SMTN	smoothelin
ENSBTAG00000046922	-0.7068	0.087651	TWIST1	twist family bHLH transcription factor 1
ENSBTAG00000024869	-0.72198	0.088135	CX3CL1	C-X3-C motif chemokine ligand 1
ENSBTAG00000034366	-0.59751	0.088135	RGS2	Bos taurus regulator of G-protein signaling 2, 24kDa (RGS2), mRNA
ENSBTAG00000020963	-0.41129	0.088135	RASSF1	ras association domain-containing protein 1
ENSBTAG00000006017	-0.31965	0.088135	NFKB2	nuclear factor NF-kappa-B p100 subunit
ENSBTAG00000031267	-0.71451	0.092811	SERPINB9	serpin family B member 9
ENSBTAG00000031707	-0.44093	0.092811	FRMD6	FERM domain containing 6
ENSBTAG00000011511	-0.43167	0.092811	novel gene	
ENSBTAG00000006703	-1.23932	0.096598	PTGDR	prostaglandin D2 receptor
ENSBTAG00000045547	-0.54536	0.097328	AMIGO3	adhesion molecule with Ig like domain 3

S1 Table

ENSBTAG00000003012	-0.43748	0.097328	TRAF1	TNF receptor associated factor 1 [
ENSBTAG00000045710	-0.86146	0.098155	novel gene	
ENSBTAG00000030182	-0.64957	0.098155	MAPK11	mitogen-activated protein kinase 11
ENSBTAG00000003018	-0.61688	0.098155	FSTL3	follistatin like 3
ENSBTAG00000021706	-0.49749	0.098155	TBX3	T-box 3
ENSBTAG00000006322	-0.45503	0.098155	DENND5A	DENN domain containing 5A
ENSBTAG00000001294	-0.40986	0.098275	PPP1R15A	protein phosphatase 1 regulatory subunit 15A
ENSBTAG00000014149	1.176536	0.000142	LCN2	lipocalin 2
ENSBTAG00000018843	1.203375	0.001792	SERPINA1	serpin family A member 1
ENSBTAG00000009725	0.509654	0.007421	AOX1	aldehyde oxidase 1
ENSBTAG00000016255	0.654815	0.007421	PLEK2	pleckstrin 2
ENSBTAG00000020657	0.427762	0.014501	FAT1	protocadherin Fat 1 precursor
ENSBTAG00000016465	0.451748	0.014501	DHCR7	7-dehydrocholesterol reductase
ENSBTAG00000020564	1.09589	0.019214	novel gene	
ENSBTAG00000001545	0.990224	0.031366	SLC34A2	solute carrier family 34 member 2
ENSBTAG00000002765	0.735138	0.032683	CYP24A1	cytochrome P450 family 24 subfamily A member 1
ENSBTAG00000015830	1.021337	0.051184	SLC5A5	solute carrier family 5 member 5
ENSBTAG00000005498	0.314534	0.057043	SQLE	squalene epoxidase
ENSBTAG00000020784	0.354524	0.057043	pseudogene	
ENSBTAG00000003140	1.018463	0.057043	CRYGS	crystallin gamma S

S1 Table

ENSBTAG00000011987	0.510289	0.065335	C14orf1	chromosome 14 open reading frame 1
ENSBTAG00000014229	0.575726	0.065335	SYT17	synaptotagmin 17
ENSBTAG00000000198	1.140032	0.065335	novel gene	
ENSBTAG00000019382	0.443074	0.066521	PKDCC	protein kinase domain-containing protein, cytoplasmic precursor
ENSBTAG00000021127	0.722708	0.074624	GNA14	G protein subunit alpha 14
ENSBTAG00000004896	0.414438	0.076909	ABLIM3	actin binding LIM protein family member 3
ENSBTAG00000037988	0.407235	0.084836	ZSCAN31	zinc finger and SCAN domain containing 31
ENSBTAG00000004990	0.316392	0.088135	NEO1	neogenin 1
ENSBTAG00000003076	0.608566	0.088135	NT5DC1	5'-nucleotidase domain containing 1
ENSBTAG00000017280	0.566862	0.09151	C3	complement C3 preproprotein
ENSBTAG00000038520	0.594617	0.092811	novel gene	
