# Supplementary material for: Interaction of preimplantation factor with the global bovine endometrial transcriptome
Source: PLoS One. 2020 Dec 7;15(12):e0242874. doi: 10.1371/journal.pone.0242874 (PMC7721156; doi:10.1371/journal.pone.0242874)
Supplement: S2 Table — Based on P adjusted values (False discovery rate: FDR; Padj<0.05) as assessed by STRING analysis. (PDF) [file pone.0242874.s004.pdf]

S2 Table. Summary of classes of KEGG pathways significantly over-represented following sPIF treatment. Based on P adjusted values (False discovery rate: FDR; *P*adj<0.05) as assessed by STRING analysis.

Class	Subclass	Number of KEGG pathways
**Human Diseases**	Infectious diseases	14
	Cancers	5
	Cardiovascular disease	1
	Endocrine and metabolic disease	1
	Immune disease	1
**Environmental Information Processing**	Signal Transduction	3
	Signalling Molecules and Interaction	1
**Organismal Systems**	Development	1
	Immune system	7
	Endocrine system	2
**Metabolism**	Lipid metabolism	1
**Cellular Processes**	Cell growth and death	2
**Genetic Information Processing**	Folding, sorting and degradation	1
