# Supplementary material for: Interaction of preimplantation factor with the global bovine endometrial transcriptome
Source: PLoS One. 2020 Dec 7;15(12):e0242874. doi: 10.1371/journal.pone.0242874 (PMC7721156; doi:10.1371/journal.pone.0242874)
Supplement: Attachment — Submitted filename: Response to Reviewers_.docx [file pone.0242874.s005.docx]

**Institute of Biological,**

**Environmental and Rural Sciences**

Penglais Campus

Aberystwyth

Ceredigion SY23 3FG

Tel: (01970) 823093

Email: rec21@aber.ac.uk

www.aber.ac.uk/en/ibers

**Athrofa y Gwyddorau Biolegol,**

**Amgylcheddol a Gwledig**

Campws Penglais

Aberystwyth

Ceredigion SY23 3FG

Ffôn: (01970) 823093

Ebost: rec21@aber.ac.uk

www.aber.ac.uk/cy/ibers

.

.

.

.

.

.

.

.

.

.

.

.

.

.

.

.

.

.

.

.

.

.

.

.

.

.

.

.

.

.

.

.

.

.

.

.

.

.

.

.

.

.

.

.

.

.

.

.

.

.

.

.

.

.

.

.

.

.

.

.

.

.

.

.

9^th^ September 2020

**Manuscript: Interaction of preimplantation factor with the global bovine endometrial transcriptome**

Dear Professor Loor,

Many thanks to you and the reviewers for assessing our manuscript titled: ‘**Interaction of preimplantation factor with the global bovine endometrial transcriptome**’. Please find a table on the following page that outlines how we have addressed and responded to all comments made by reviewers in detail. The new ‘revised highlighted’ manuscript uploaded indicates where these changes have been made in tracked changes.

We hope these amendments address the reviewers’ comments and enable the manuscript to proceed to being accepted. We welcome your response with thanks. Thank you for your time in considering the present paper. We look forward to hearing your decision in the near future.

Yours sincerely,

Ruth Wonfor (R. E. Wonfor) Ph.D

Lecturer in Animal and Equine Science,

Institute of Biological, Environmental and Rural Sciences (IBERS),

**Manuscript Number**: PONE-D-20-09982

**Title**: Interaction of preimplantation factor with the global bovine endometrial transcriptome

**Authors**: Wonfor RE, Creevey, CJ, Natoli M, Hegarty, M, Nash DM, Rose MT

**Response to Reviewers’ Comments**

The authors would like to express our sincere thanks to the editor and the reviewers for taking the time to read our manuscript and for helping us to make it better – your work is very much appreciated.

**Reviewer #1**

| **Comment** | **Response (Line numbers refer to the revised highlighted manuscript)** |
| --- | --- |
| All comments have been addressed properly and most of necessary changes have been made in relation with present possibilities for making them. | We thank the reviewer for your time assessing the revisions and are pleased to hear that we have addressed your comments properly. |

**Reviewer #2**

| **Comment** | **Response (Line numbers refer to the revised highlighted manuscript)** |
| --- | --- |
| L80 currently reads “..differences in maternal recognition and early pregnancy in humans compared to cattle”. I suggest changing this to “differences in the maternal recognition of pregnancy and the timing and mode of implantation… | Line 80-81: amended as indicated |
| The description of the explant methodology is improved. One further point, if the explants weighed on average 42 mg but <20 mg was used to extract, presumably a piece was cut off prior to extraction. Please explain. | Line 131: Amended to make clear that <20 mg was cut off each explant that RNA was extracted from whilst still frozen, using sterile scissors. |
| Lines 236-238. The accuracy of the variation calculation from the PCA plots does not justify giving the outcomes to 2 decimal places. | Lines 236-238: Amended to 1 decimal place in the text. |

**Reviewer #3**

| **Comment** | **Response (Line numbers refer to the revised highlighted manuscript)** |
| --- | --- |
| On lines 193 and 198 the authors indicated: “the B. taurus genome used as the statistical background”  A critical aspect of in silico analysis of gene function is the choice of genes to compose the background list. See papers listed below for a reference as to how using the wrong list of genes, such as all genes in a given genome, can produce biased results. This problem must be addressed. | The data have been reanalysed in String v11.0 using the genes used in the DESeq analysis as the statistical background. As a result of this there were some changes in P values and genes included in KEGG pathways. Furthermore, there were more KEGG pathways over-represented with DEG. As such, the following amendments have been made:   - Line 29: abstract updated - Line 193 and 197, String version changed. - Line 193: change in the statistical background definition. - Line 281-292: amended Go analysis section to reflect re-analysis - Line 294-338: amended KEGG analysis section to reflect re-analysis, including updating Table 2 and S2 Table and amending Fig2 to reflect genes included in new analysis. - Line 409-430: added in reference to IL-17 and MAPK signalling pathways to further support the results. - Lines 341-357 and Fig 4 amended to reflect updates to String (reanalysed in version 11 to match the KEGG and GO category analysis). However, it is not possible with STRING to generate a protein interaction network with the DEseq analysis gene list. I have queried this with STRING and received the following explanation from Damian Szklarczyk: “If you do not input the background yourself, the background is assumed to be the whole STRING proteome. Because it is the default, we have pre-computed all the necessary numbers, and different combinations of cut-off and channels (2504 combinations per species). Then we compare it to the distribution of links within your input and compare these two to generate the p-value. If you provide your own background we would have to recompute the background distribution of PPI, each time your query, which would take a lot of resources. The cut-off below we compute it is 8000 proteins.”   Therefore, with our background of >15,000 proteins, we are unable to use this to assess the protein interaction network. “ |
